# Interactive Effects of Dietary Fat/Carbohydrate Ratio and Body Mass Index on Iron Deficiency Anemia among Taiwanese Women

**DOI:** 10.3390/nu6093929

**Published:** 2014-09-24

**Authors:** Jung-Su Chang, Yi-Chun Chen, Eddy Owaga, Khairizka Citra Palupi, Wen-Harn Pan, Chyi-Huey Bai

**Affiliations:** 1 School of Nutrition and Health Sciences, College of Public Health and Nutrition, Taipei Medical University, 250 Wu-Hsing Street, Taipei 110, Taiwan; E-Mails: susanchang@tmu.edu.tw (J.-S.C.); yichun@tmu.edu.tw (Y.-C.C.); khairizka.citra@gmail.com (K.C.P.); 2 Institute of Food and Bioresources Technology, Dedan Kimathi University of Technology, Nyeri 10100, Kenya; E-Mail: eowaga@yahoo.co.uk; 3 Institute of Biomedical Science, Academia Sinica, 128 Sec. 2, Academia Rd. Nankang, Taipei 115, Taiwan; E-Mail: pan@ibms.sinica.edu.tw; 4 Department of Public Health, College of Medicine, Taipei Medical University, 250 Wu-Hsing Street, Taipei 110, Taiwan

**Keywords:** iron deficiency anemia, dietary fat and carbohydrate, overweight and obesity, Taiwanese female

## Abstract

Whether being overweight or obese is associated with increased risk of iron deficiency anemia (IDA) remains controversial. We evaluated the dietary intakes and risk for IDA in relation to body mass index (BMI). One thousand two hundred and seventy-four females aged ≥19 years, enrolled in the third Nutrition and Health Survey in Taiwan (NAHSIT) 2005–2008, were selected. Half of the women were either overweight (24.0%) or obese (25.3%). The overall prevalence of anemia, iron deficiency and IDA among adult women was 19.5%, 8.6% and 6.2%. BMI showed a protective effect on IDA: overweight (odds ratio, OR: 0.365 (0.181–0.736)) and obese (OR: 0.480 (0.259–0.891)) when compared with normal weight. Univariate analysis identified increased IDA risk for overweight/obese women who consumed higher dietary fat but lower carbohydrate (CHO) (OR: 10.119 (1.267–80.79)). No such relationship was found in IDA women with normal weight (OR: 0.375 (0.036–4.022)). Analysis of interaction(s) showed individuals within the highest BMI tertile (T3) had the lowest risk for IDA and the risk increased with increasing tertile groups of fat/CHO ratio; OR 0.381 (0.144–1.008; *p* = 0.051), 0.370 (0.133–1.026; *p* = 0.056) and 0.748 (0.314–1.783; *p* = 0.513); for T1, T2 and T3, respectively. In conclusion, a protective effect of BMI on IDA may be attenuated in women who had increased fat/CHO ratio.

## 1. Introduction

A possible relationship between obesity and hypoferremia has recently been described in children [1,2,3] and women [4,5,6,7,8,9,10]. However, whether being overweight or obese is associated with increased risk of anemia or iron deficiency anemia (IDA) is still being debated [7,8,9,10,11,12,13,14]. Cheng and colleagues conducted a systematic review of 25 studies investigating iron status in obese populations and reported obese people tended to have higher hemoglobin (Hb) and ferritin concentrations, but lower transferrin saturation compared to non-obese controls [15]. This study supports the view that hypoferremia is associated with obesity, but the higher Hb levels observed in overweigh/obese indicates that sufficient iron is available for erythropoiesis [15].

Hypoferremia is defined as low serum iron concentrations and is a common response to systemic infection or chronic inflammation [16]. Hypoferremia during infections has been suggested as one of the host defense mechanisms against infections by limiting iron availability for pathogens. Obesity-related hypoferremia, on the other hand, is in part due to low-grade inflammation. Inflammatory mediators such as interleukin-6 (IL-6) and interleukin-10 (IL-10) play a key role in iron homeostasis. IL-6 promotes the development of obesity-related hypoferremia by targeting the hepcidin-ferroportin-1 axis [17]. Hepcidin, a negative regulator of iron absorption and iron release, is positively correlated with the body mass index (BMI) but inversely correlated with serum iron concentrations [3]. Thus, the consensus is that elevated hepcidin levels promote the development of obesity-related hypoferremia. However, it is difficult to conclude what effect the degree of obesity has on hepcidin levels and whether elevated hepcidin levels directly contribute to the development of anemia or IDA.

Another possible mechanism linking obesity to hypoferremia is diet. Overweight/obese people may develop paradoxical nutritional deficiency from eating high-energy (e.g., protein and fat) but low micronutrient foods (e.g., vitamins and trace elements) [18,19]. In underweight individuals, micronutrient deficiency, especially iron deficiency or dietary iron absorption-regulating factors (e.g., vitamin C and copper) or iron distribution factors (e.g., vitamin A), are believed to be the main underlying cause of hypoferremia or IDA. However, some studies investigating the association between dietary factors and obesity-related hypoferremia showed a poor correlation between serum iron concentrations and dietary iron intake in obese/overweight people [4,5,6]. These studies suggest that, unlike studies in normal weight or underweight people, micronutrient deficiency may not be the primary cause of obesity-related hypoferremia.

Nutritional risk factors for IDA are often reported in underweight people. Overweight/obese subjects have altered foods choices and metabolic demand; therefore, this knowledge may not apply to overweight/obese people. The aim of this study was to investigate the separate and interactive effects of dietary components and BMI on iron status in women enrolled in Nutrition and Health Survey in Taiwan (NAHSIT 2005–2008, Adults). The specific aims of this study were as follows: (1) to assess the relationship between iron status and BMI; (2) to identify the dietary components that associate with iron status in relation to BMI; and (3) to examine the interactive effects of dietary components and BMI on IDA.

## 2. Experimental Section

### 2.1. Study Design

The Third National Nutrition and Health Survey in Taiwan (NAHSIT) 2005–2008 was funded by the Department of Health to provide continued assessment of health and nutrition of the people in Taiwan. The survey was conducted using a multi-staged, stratified and clustered sampling scheme which includes a wide range of age groups across the whole of Taiwan [20,21]. The present study only analyzed data obtained from females aged ≥19 years. This study was approved by the Research Ethics Committee of Taipei Medical University (201203029) and Academia Sinica (AS-IRB01-07020). Written informed consent was obtained from all the participants.

### 2.2. Data Collection

Initially, a total of 1348 female participants were recruited to this study. Exclusion criteria were as follows: (1) individuals with missing data for iron biochemistry, anthropometry and 24 h dietary recall; (2) total calorie intake ≥5000 kcal/day or ≤500 kcal/day; (3) fat/carbohydrate (CHO) ratio <1% and >99%; and (4) medical history of cancer, chronic liver diseases (e.g., hepatitis, liver fibrosis/cirrhosis) and chronic kidney diseases. As such, a total of 1274 female participants were enrolled in the study. Information on family health history and 24 h dietary recall were obtained using a standardized questionnaire. Measurements of body weight and height, waist circumference and blood pressure were described as elsewhere [22]. Dietary intake was estimated by 24 h dietary recall which includes measurement of household recipes, the individual dietary recall and validation of the recall using food models. Total caloric intake, total iron intake, type of iron (heme iron and non-heme iron) consumed, intakes of carbohydrates, protein, fats and oils were obtained from the 24 h dietary recall [23,24]. All nutrient intakes were energy adjusted by the residual method [25]. Details on the data collection and analysis has been described elsewhere [23,24].

### 2.3. Definitions of Iron Deficiency Anemia (IDA)

Biochemistry data were obtained from 8 h-fasting blood samples. Heparinized whole blood was collected for on-site measurement of Hb. Iron status was evaluated by serum iron, transferrin saturation and serum ferritin concentrations [26]. Serum ferritin was measured using electrochemiluminescence immunoassay and was quantitated by the Roche Modular P800. Hb was measured by the cyanomethaemoglobin method (Merckotest, Merck, Darmstadt, Germany) using a portable filter photometer calibrated with Hb cyanide standard solution (Merck, Darmstadt, Germany). Serum iron and total iron binding capacity (TIBC) were measured by ferrozine-based colorimetric method. Percentage transferrin saturation (TS) was calculated by serum iron/TIBC × 100%. Iron deficiency was considered if ≥2 indicators of iron status were abnormal: Serum ferritin < 12 ng/mL, transferrin saturation < 15% and Hb < 12 g/dL. Anemia was considered if Hb < 12 g/dL in female. Iron overload was defined as serum ferritin > 200 ng/mL in female.

### 2.4. Definitions of Overweight and Obesity

Obesity and overweight were defined based on definitions used by the Department of Health in Taiwan [27,28]. This definition is in accordance with the World Health Organization (WHO)-Asian’s criteria which define overweight as BMI ≥ 24 kg/m^2^ and obese as BMI ≥ 27 kg/m^2^ [29].

### 2.5. Statistical Analyses

Statistical analyses were performed using the Statistical Analysis Systems software (SAS version 9.22; SAS Institute, Taipei, Taiwan). Categorical data was presented as number (percentage) while continuous data presented as mean (standard deviation). The one-way analysis of variance and chi-square test were used to compare the differences among body weight groups. Logistic regression models were used to estimate the OR and 95% confidence interval (CI) for IDA risk. To further analyze the relationship between fat/CHO ratio, BMI and risk of IDA, a binary logistic model was employed. In Table 1, trend test was carried out by ordinal chi-square test or by linear regression for continuous data. In Table 2, the underweight females (BMI <19 kg/m^2^) (*n* = 103) were excluded from analysis. All nutrient intakes were adjusted by total energy using the residual method. *p* < 0.05 was considered statistically significant.

## 3. Results

### 3.1. Association between BMI and Iron Status

The mean age of study participants was 52.9 ± 0.5 years old and mean BMI was 24.4 ± 0.1 kg/m^2^. Five hundred forty-three (42.6%) women were classified as normal body weight while 103 (8.1%) women were diagnosed as underweight (Table 1). Half of the women were either overweight (24.0%) or obese (25.3%). Two hundred eighty-seven (25.4%) women were diagnosed as having metabolic syndrome (MetS). Women with MetS had higher mean serum ferritin levels compared with healthy individuals; 172 (4.2) ng/mL and 120 (2.6) ng/mL, respectively. Compared with women with normal metabolic status, women with MetS had a significantly higher rate of iron overload (23.7% *versus* 76.3%). The overall prevalence of anemia, iron deficiency and IDA among Taiwanese adult women was 19.5%, 8.6% and 6.2%; respectively. The menopausal status was closely related to the prevalence of IDA (11.6% for premenopausal women and 1.0% for postmenopausal women) but not anemia (20.5% for premenopausal women and 18.3% for postmenopausal women) (data not shown). No association between serum iron concentrations and BMI was found (Table 1). However, positive relationships between BMI and serum ferritin and Hb levels were observed (all *p* < 0.0001). Negative relationships between BMI and prevalence of iron deficiency and IDA were found (*p* = 0.04 and *p* = 0.002). In a univariate analysis, BMI showed a protective effect on IDA: overweight (OR: 0.365 (0.181–0.736)) and obese (OR: 0.480 (0.259–0.891)) when compared with normal body weight (data not shown). These data suggest that being overweight or obese did not associate with increased risk of IDA.

**Table 1:** Relationship between iron status and body mass index in female aged ≥19 years (*n* = 1274).

Variables	Underweight ^a^	Normal Weight ^a^	Overweight ^a^	Obese ^a^	*p*-trend
Number (*n*, %)	103.0 (8.1)	543.0 (42.6)	306.0 (24.0)	322.0 (25.2)	
Age (years)	39.3 (1.9)	49.9 (0.8)	57.2 (0.9)	56.4 (0.9)	<0.0001
Waist (cm)	64.7 (0.5)	74.5 (0.3)	83.5 (0.4)	93.0 (0.5)	<0.0001
BMI (kg/m^2^)	17.8 (0.1)	21.6 (0.06)	25.4 (0.04)	30.1 (0.1)	<0.0001
Serum iron (μg/dL)	99.9 (4.5)	96.4 (1.8)	97.2 (2.2)	94.6 (1.9)	0.2654
Serum ferritin (ng/mL)	55.4 (5.1)	89.2 (3.6)	120.6 (5.7)	120.4 (5.6)	<0.0001
Hemoglobin (g/dL)	12.5 (0.1)	12.6 (0.1)	12.9 (0.1)	13.0 (0.1)	<0.0001
Anemia (*n*, %)	23.0 (22.3)	124.0 (22.8)	54.0 (17.6)	50.0 (15.5)	0.0280
Iron depletion (*n*, %) ^b^	54.0 (52.4)	285.0 (52.4)	158.0 (51.6)	171.0 (53.1)	0.9669
Iron deficiency (*n*, %) ^c^	15.0 (14.5)	55.0 (10.1)	20.0 (6.5)	20.0 (6.2)	0.0414
Iron deficiency anemia (*n*, %) ^d^	11.0 (10.6)	45.0 (8.2)	10.0 (3.2)	14.0 (4.3)	0.0024

^a^ Weight categories: underweight: BMI < 19 kg/m^2^, normal weight: ≥19–23 kg/m^2^, overweight: 24 kg/m^2^ ≤ BMI < 27 kg/m^2^ and obese: BMI ≥ 27 kg/m^2^; ^b^ Iron depletion was considered if any of the two iron indicators showed abnormal values: SF ˂ 20 ng/mL and %TS < 30%; ^c^ Iron deficiency was considered if both iron indicators showed abnormal values: SF ˂ 12 ng/mL and %TS < 15%; ^d^ Iron deficiency anemia (IDA): SF ˂ 12 ng/mL, TS < 15% and Hb < 12 g/dL.

### 3.2. Association between Dietary Components, BMI and Iron Deficiency Anemia

Literature indicates that foods selected by overweight/obese women may differ from those of normal weight women. Therefore, we next investigated the relationship between dietary factors, IDA and BMI. Initially, a total of 88 women were diagnosed with IDA. 8 IDA women were excluded from further analysis due to invalid information on dietary intakes. We also excluded 11 IDA women who were underweight. As such, a total of 69 IDA women were selected for the analysis. Table 2 shows overweight/obese IDA women were slightly older with lower prevalence of IDA than those with normal weight (*p* < 0.0001). Compared with normal weight IDA women, overweight/obese IDA women were more likely to consume higher amounts of dietary fat (*p* = 0.0591), and lower amounts of carbohydrate (*p* = 0.0457), resulting in increased fat/CHO ratio (*p* = 0.0171). In a univariate analysis, age showed a slight protective effect on IDA for both normal weight and overweight/obese; 0.974 (0.955–0.993) and 0.951 (0.926–0.976); respectively (Table 2). Univariate analysis identified increased IDA risk for overweight/obese women who consumed higher dietary fat and lower CHO (OR: 10.119 (1.267–80.79)). No such relationship was found in IDA women with normal body weight (OR: 0.375 (0.036–4.022)).

**Table 2:** Relationships between dietary intake, body mass index and iron deficiency anemia (*n* = 1072).

Variables	Iron Deficiency Anemia						
	Normal Weight Women ^a^			Overweight/Obese Women ^b^			
	IDA (−)	IDA (+)	ORs (95% CI) ^c^	IDA (−)	IDA (+)	*p*–value ^d^	ORs (95% CI) ^c^
Number (*n*)	446.0	45.0		557.0	24.0		
Age (years)	50.0 (0.8)	42.6 (2.03)	0.974 (0.955–0.993)	57.1 (0.7)	44.5 (1.8)	<0.0001	0.951 (0.926–0.976)
BMI (kg/m^2^)	21.7 (0.06)	21.3 (0.21)	0.830 (0.660–1.043)	27.8 (0.2)	27.9 (0.6)	<0.0001	1.011 (0.896–1.140)
**Nutritional Intake ^e^**							
Protein (g/day)							
all	69.0 (1.0)	70.4 (3.1)	1.004 (0.988–1.019)	66.6 (1.0)	64.3 (4.6)	0.3805	0.996 (0.980–1.012)
plant	33.5 (0.8)	32.5 (2.6)	0.995 (0.975–1.015)	31.9 (0.7)	28.3 (3.3)	0.4036	0.987 (0.962–1.013)
animal	32.2 (1.1)	34.9 (3.4)	1.006 (0.992–1.020)	32.3 (1.0)	34.3 (4.8)	0.9861	1.003 (0.988–1.017)
Fat (g/day)	54.8 (1.0)	51.0 (3.1)	0.990 (0.974–1.007)	54.4 (1.0)	60.0 (4.7)	0.0591	1.010 (0.993–1.027)
Carbohydrate (g/day)	222.0 (2)	230.0 (8)	1.003 (0.996–1.009)	212.0 (2)	198.0 (11)	0.0457	0.996 (0.990–1.003)
Fat/CHO ratio	0.256 (0.007)	0.234 (0.018)	0.375 (0.036–4.022)	0.260 (0.007)	0.335 (0.046)	0.0171	10.119 (1.267–80.797)
Iron (mg/day)							
all	14.8 (0.6)	14.7 (2.0)	0.998 (0.971–1.027)	13.3 (0.4)	12.6 (1.7)	0.4919	0.991 (0.943–1.041)
plant	11.1 (0.6)	10.8 (1.9)	0.997 (0.967–1.027)	10.0 (0.3)	8.5 (1.6)	0.4104	0.972 (0.911–1.037)
animal	3.4 (0.2)	3.6 (0.6)	1.013 (0.940–1.091)	3.1 (0.1)	3.9 (0.7)	0.7126	1.041 (0.960–1.129)
Plant/animal	12.9 (01.7)	4.6 (5.5)	0.955 (0.904–1.008)	12.7 (1.8)	8.1 (8.6)	0.7644	0.993 (0.966–1.020)
Fiber (mg/day)	17.9 (0.6)	17.8 (1.9)	0.998 (0.971–1.026)	16.8 (0.5)	12.7 (2.2)	0.0991	0.959 (0.913–1.008)
Vit B 6 (mg/day)	1.67 (0.04)	1.63 (0.13)	0.948 (0.647–1.390)	1.67 (0.04)	1.31 (0.18)	0.1988	0.586 (0.315–1.089)
Vit B 12 (μg/day)	6.1 (0.9)	5.3 (2.7)	0.996 (0.970–1.022)	5.2 (0.4)	3.7 (2.2)	0.6834	0.988 (0.936–1.044)
Vit C (mg/day)	177 (7)	197 (22)	1.001 (0.999–1.003)	166 (6)	173 (30)	0.5646	1.000 (0.998–1.003)
Vit D (μg/day)	6.9 (0.6)	7.4 (1.9)	1.002 (0.979–1.026)	8.2 (0.5)	4.0 (2.5)	0.3068	0.951 (0.892–1.014)

^a^ Normal weight women: 19 kg/m^2^ ≤ BMI < 24 kg/m^2^; ^b^ Overweight: 24 kg/m^2^ ≤ BMI <27 kg/m^2^ and obesity: BMI ≥ 27 kg/m^2^; ^c^ Univariate analysis: IDA as a dependent variable; ^d^ *p*-value: normal weight women with IDA (+) compared with overweight/obese women with IDA (+); ^e^ Nutrient intakes were energy adjusted by the residual method.

### 3.3. Interactive Effects of Dietary Fat/Carbohydrate Ratio and BMI on Iron Deficiency Anemia

To further classify the relationship between fat/CHO ratio, BMI and risk of IDA, categorical logistic model was employed. Table 3 shows individuals within the highest BMI tertile (T3) had the lowest risk for IDA and the risk increased with increasing tertile groups of fat/CHO ratio; OR 0.381, 0.370 and 0.748; for T1, T2 and T3 respectively (Table 3, Crude). The OR became weaker after further adjustment for age (Table 3, Age-adjusted).

**Table 3:** Interactive effects of dietary fat/carbohydrate ratio and body mass index on iron deficiency anemia (*n* = 1274).

Fat/CHO Ratio ^b^	BMI ^a^					
	T1	*p*-value	T2	*p*-value	T3	*p*-value
**Crude**						
T1	Reference	-	0.915 (0.354–2.363)	0.853	0.381 (0.144–1.008)	0.051
T2	1.402 (0.582–3.380)	0.451	1.008 (0.390–2.610)	0.986	0.370 (0.133–1.026)	0.056
T3	1.519 (0.615–3.753)	0.364	1.123 (0.433–2.915)	0.811	0.748 (0.314–1.783)	0.513
**Age Adjusted**						
T1	Reference	-	1.202 (0.454–3.181)	0.711	0.547 (0.201–1.487)	0.236
T2	1.093 (0.446–2.679)	0.841	1.038 (0.396–2.725)	0.939	0.488 (0.172–1.382)	0.176
T3	1.016 (0.403–2.565)	0.972	1.114 (0.423–2.936)	0.827	0.798 (0.330–1.927)	0.615

^a^ BMI tertile: 22.1/25.8 kg/m^2^; ^b^ Fat/carbohydrate tertile: 0.1689/0.2916.

Table 4 shows fat/CHO ratio were closely associated with BMI and this trend was only observed in IDA women and not healthy women. Among IDA women, a positive relationship between BMI and fat consumption (*p* = 0.035) and an inverse relationship between BMI and CHO intake (*p* = 0.045) were observed. In contrast, a negative association was found between iron intake and BMI in healthy women (*p* = 0.001).

## 4. Discussion

To our knowledge, this cross-sectional, population-based study is the first to clarify the association between BMI, nutritional risk factors and IDA in Chinese women. Our results support the view that being overweight or obese is not associated with increased risk of iron deficiency or IDA. Both BMI and age were associated with reduced risk for IDA. This suggests that reproductive-aged women who were underweight or normal weight were more likely to develop IDA than post-menopausal overweight/obese women. Our results also raise the possibility that nutritional risk factors for IDA may differ between normal weight and overweight/obese women. Overweight/obese women who consumed high fat/low CHO diet were 10.11 (1.267–80.797) times more likely to develop IDA. The protective effect of BMI on IDA was reduced when overweight/obese women increased fat/CHO ratio.

**Table 4:** Association between dietary intakes, body mass index and iron deficiency anemia (*n* = 1274).

Variables	IDA (−)				IDA (+)			
	BMI ^a^				BMI ^b^			
**Energy Adjusted ^c^**	**T1**	**T2**	**T3**	***p*-trend**	**T1**	**T2**	**T3**	***p*-trend**
Fat (g/day)	54.3 (1.2)	53.2 (1.2)	56.0 (1.1)	0.281	50.7 (4.4)	55.8 (4.5)	63.8 (4.3)	0.035
Carbohydrate (g/day)	218 (3)	220 (3)	212 (3)	0.126	237 (11)	222 (11)	206 (11)	0.045
Protein (g/day)	69.0 (1.2)	67.4 (1.2)	67.0 (1.1)	0.196	68.9 (4.3)	76.0 (4.4)	67.9 (4.2)	0.871
Animal/Plant	1.29 (0.07)	1.33 (0.07)	1.31 (0.07)	0.805	1.11 (0.24)	1.56 (0.25)	1.34 (0.23)	0.486
Fat/Carbohydrate	0.27 (0.01)	0.25 (0.01)	0.28 (0.01)	0.505	0.24 (0.03)	0.25 (0.03)	0.33 (0.03)	0.041
Fat/Protein	0.81 (0.02)	0.80 (0.02)	0.84 (0.02)	0.337	0.78 (0.08)	0.74 (0.08)	0.98 (0.08)	0.091
Iron (mg/day)	15.9 (0.6)	13.2 (0.6)	13.2 (0.6)	0.001	13.3 (1.5)	16.8 (1.5)	13.2 (1.4)	0.954
Animal Fe/Plant Fe	0.51 (0.03)	0.45 (0.03)	0.46 (0.03)	0.245	0.43 (0.12)	0.56 (0.12)	0.53 (0.11)	0.544
Plant Fe/Vit C	0.13 (0.02)	0.10 (0.02)	0.13 (0.02)	0.968	0.09 (0.10)	0.12 (0.10)	0.24 (0.10)	0.275
Vit B6 + B12	8.75 (0.78)	6.47 (0.78)	6.90 (0.77)	0.094	5.79 (1.53)	8.12 (1.57)	5.63 (1.49)	0.941

^a^ BMI cutpoint for female without IDA (−): 22.2/26.0 kg/m^2^; ^b^ BMI cutpoint for female with IDA (+): 20.5/23.3 kg/m^2^; ^c^ Nutrient intakes were energy adjusted by the residual method.

Our study indicates that obesity-related IDA may be explained by differences in food choices, particularly macronutrients and not micronutrients. Overweight/obese IDA women tended to replace CHO with fat as sources of energy compared with normal weight IDA women (normal weight: 56% energy from CHO and 27.5% energy from fat; overweight/obese: 48% energy from CHO and 34.4% energy from fat). Animal studies show high fat [30,31] or high fat and high fructose diet [32] induced altered iron metabolism and this is in part due to the abnormal hepcidin levels. Upon administration of radioactively labeled ^59^ferric citrate into mice, Sonnweber *et al.* reported that the high fat diet-induced hypoferremia is due to the diminished intestinal iron uptake [30]. However, mice fed with high fat and high fructose diet for 16 weeks developed hepatic iron accumulation suggesting dysfunctional hepatic iron release [32].

In animal studies, a high fat diet normally contains 60% energy from fat, which is unlikely to occur in humans. In humans, overweight/obese people tend to replace CHO with fat and protein as a source of energy [24]. Although our study observed no risk association between protein intakes and IDA, we can’t rule out a possibility of its interaction with iron metabolism [23]. By comparing the two consecutive Nutrition and Health Surveys involving Taiwanese adults (NAHSIT 1993–1996 *vs.* 2005–2008), Wu and colleagues reported highest intake of animal protein and fat among young Taiwanese adults aged 19–30 years compared with other age groups. Animal proteins, particularly red meat, are rich sources of heme iron. Our study found that average iron intake in Taiwanese adult women is 15.2 mg/day, reaching 100% dietary recommended intake for iron for reproductive-aged women. The average iron intake did not differ significantly between anemic and non-anemic women (13.8 *vs.* 14.2 mg/day); nor did normal weight and overweight/obese women (14.6 *vs.* 13.9 mg/day). The average iron intake is slightly lower in overweight/obese IDA women than in normal weight IDA women; 14.7 *vs.* 12.6 mg/day; respectively. This is due in part to a reduction in plant iron intake (normal weight IDA women: 10.8 and overweight/obese: 8.5 mg/day) and not animal iron intake (normal weight: 3.6 and overweight/obese: 3.9 mg/day). This results in an increased animal/plant iron ratio in overweight/obese IDA women compared with normal weight IDA women (0.48 *vs.* 0.33). Overall, our study suggests that dietary iron deficiency may not be the primary cause of obesity-related IDA in adult women. However, future research may be needed to clarify whether replacing CHO by fat as sources of energy have long-term effect on intestinal iron uptake and intracellular iron release in human.

There are several limitations in our study, which need to be taken into account during interpretation of the results. Our study is limited by the small sample size with a low prevalence rate of IDA (6.2%). It is also confined by the nature of a cross-sectional study. In order to establish the casual relationship between fat/CHO intake, BMI and IDA, longitudinal studies are necessary so as to understand if changes in fat/CHO intake over time can predict disease susceptibility in overweight/obese women. The use of 24 h dietary record to obtain reliable data on nutritional intakes may be limited by the insufficient time period for data collection. Hence, an eight-day dietary record has been suggested as the minimum number of record days to determine macronutrient in order to minimize the effect of random error [33]. Jackson and colleagues reported fat and proteins intakes were significantly more variable than CHO on Sundays compared to weekdays, for both men and women [33]. Our study found a great variation in fat intakes compared with CHO. The median fat intake was 44.8 g/day among adult female. However, 1% female consumed an average 3.9 g fat per day and the upper 99% female reported an average 195 g/day fat intake. Due to the large variation in fat intakes, our study excluded women with fat intake <1% and >99% from analysis. Underreporting of food intake is common in obese subjects. It has been shown that obese individuals may selectively underreport fat intake [34]. Due to a wide variation in geographical location and limitation in man power, our NAHSIT survey for dietary intake was limited to 24 h.

## 5. Conclusions

Our study of Chinese adult females showed that reproductive-aged women who were underweight or of normal weight were more likely to develop IDA compared with overweight/obese women. The protective effect of BMI on IDA may be attenuated in women who consume high fat but low CHO diet.
